# Acute Febrile Encephalopathy with Rigidity

**DOI:** 10.1177/08850666251345448

**Published:** 2025-05-30

**Authors:** Dearbhla M. Kelly, Eoin M. Kelleher

**Affiliations:** 1 Oxford Critical Care, John Radcliffe Hospital, University of Oxford, Oxford, UK; 2 Wolfson Centre for the Prevention of Stroke and Dementia, Nuffield Department of Clinical Neurosciences, John Radcliffe Hospital, University of Oxford, Oxford, UK; 3 Nuffield Department of Clinical Neurosciences, University of Oxford, Oxford, UK

**Keywords:** neuroleptic malignant syndrome, rigidity, hyperpyrexia, serotonin syndrome, malignant hyperthermia, parkinsonism-hyperpyrexia syndrome

## Abstract

Febrile encephalopathy with rigidity can be the presenting feature of several life-threatening neurological emergencies such as neuroleptic malignant syndrome (NMS) and serotonin syndrome. Although the pathogenesis of these conditions is poorly understood, we review advances in our understanding of underlying genetic and other biological mechanisms. We also discuss the complex and expanding differential diagnoses which include other drug-induced hyperpyrexia or rigidity syndromes (eg malignant hyperthermia, parkinsonism-hyperpyrexia syndrome, malignant catatonia) as well as autoimmune syndromes. Finally, we consider potential predictive and preventative approaches along with best practice management strategies.

## Introduction

The clinical syndrome of acute febrile rigidity with dysautonomia encompasses a myriad of conditions including neuroleptic malignant syndrome (NMS), serotonin syndrome, and malignant hyperthermia. Distinguishing between these drug-induced hyperpyrexia or rigidity syndromes can be challenging with overlapping symptoms and signs seen in several cases. In this review, we will describe the current epidemiology, pathogenesis, diagnosis and management of these conditions with a focus on advances in our understanding of the underlying biological mechanisms, risk stratification, and preventative measures.

## Neuroleptic Malignant Syndrome

### Epidemiology

NMS is a rare and life-threatening idiosyncratic reaction that most commonly occurs following exposure to antipsychotic medications. It was initially described as ‘fatal hyperpyrexia’ in 1956 when it was first reported by Frank J. Ayd, shortly after the introduction of the first-generation antipsychotic agent chlorpromazine.^1^ There are reported incidence rates of between 0.2–3% among antipsychotic users with the wide range likely reflecting differences in the study settings or in the disease definition used.^2,3^ It affects all ages but is most commonly described in young men as the most frequent users of neuroleptics.^4^

### Risk Factors

NMS is primarily triggered by the blockade of dopamine receptors, with antipsychotic medications being the usual cause. High-potency typical antipsychotics, including haloperidol, trifluoperazine, and prochlorperazine, are most commonly associated with NMS.^5^ These medications are considered the highest risk for NMS due to their greater affinity for dopamine D2 receptors and slow dissociation.^6^ While some studies indicate that the incidence has decreased with the advent of atypical antipsychotics,^7,8^ NMS has still been observed with low-potency (eg, chlorpromazine) and second-generation antipsychotics (eg, clozapine, olanzapine).^9,10^ Although the syndrome is mainly triggered by antipsychotics, it can rarely be provoked by other dopamine (D2) receptor antagonists, such as anticonvulsants, antidepressants, and metoclopramide.^11,12^

While NMS can unpredictably occur at any point during drug treatment, it is more commonly observed within the first month of therapy.^13^ Recent, rapid or high dose escalation, drug change, and parenteral administration have also been cited as additional pharmacological risk factors.^4,14,15^ A previous episode of NMS or a personal and/or family history of catatonia also increases the risk of developing NMS.^16^ Environmental factors implicated as potentially contributory include physical restraint, elevated ambient temperature, and volume depletion,^17^ likely related to their impact on an already disorderly thermoregulation. There are also a few case reports of NMS occurring with antipsychotic use in the context of Covid-19 infection.^18,19^ However, these were likely coincidental given the extent of the epidemic at the time.

#### Pathogenesis

Although the exact pathobiology of NMS is unknown, there are two main postulated hypotheses (Figure 1). In the first hypothesis, blockade of central dopamine receptors in the hypothalamus and disruption of the nigrostriatal dopamine pathways are implicated.^20^ Dopamine neurotransmission plays an important role in thermoregulation, particularly in the anterior pre-optic nucleus of the hypothalamus.^21^ Central dopaminergic D2 receptor antagonism via medications such as antipsychotics is thought to trigger hyperpyrexia and other signs of dysautonomia.^20,22^ Altered dopamine signalling in the basal ganglia where motor coordination and muscle tone are regulated may account for some of the extrapyramidal features of NMS such as muscular rigidity and tremor. However, there is evidence to suggest that other neurotransmitter systems such as catecholamines or serotonin may also play a role.^23^

**Figure 1.**
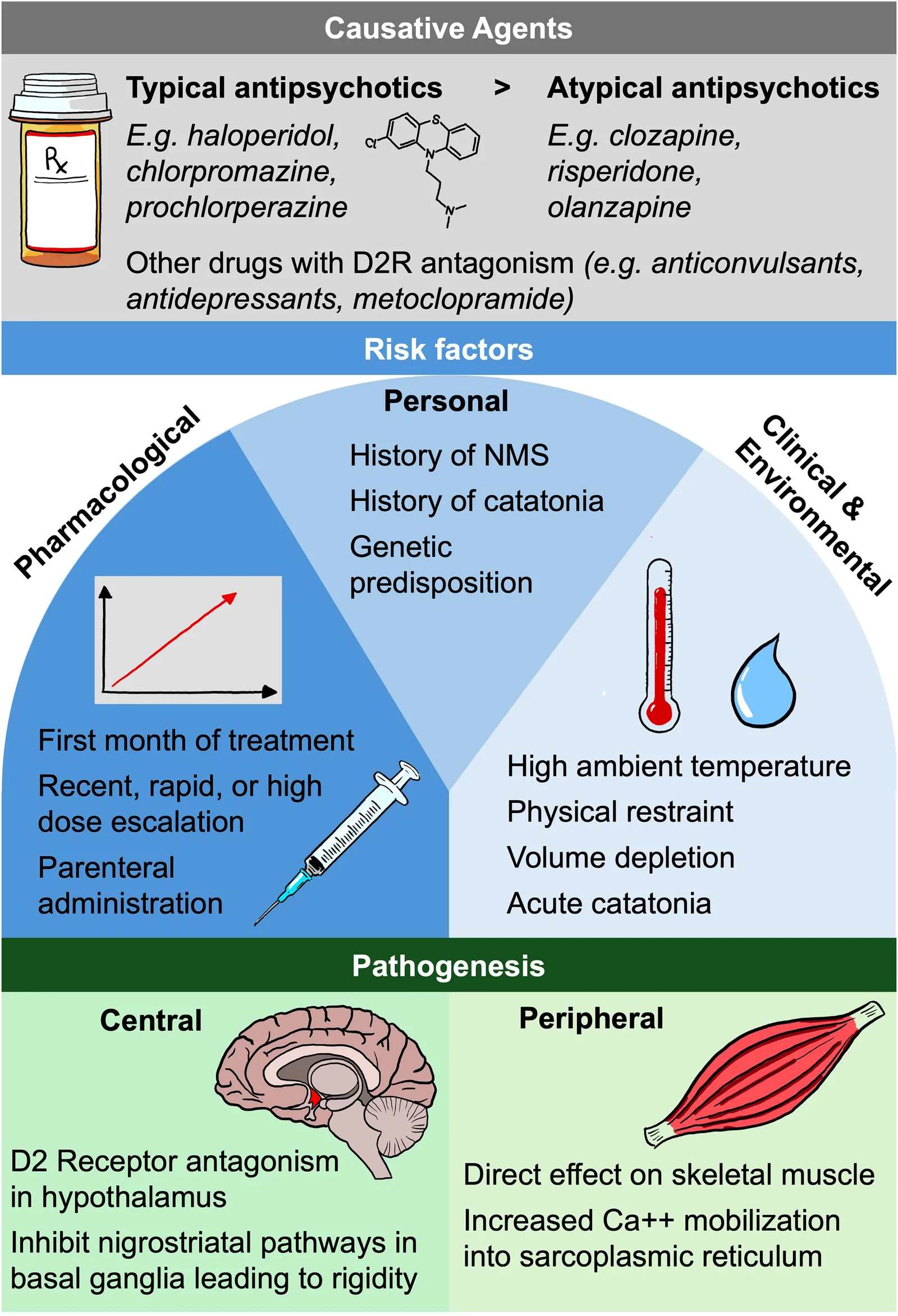
Current Theories of Neuroleptic Malignant Syndrome Pathophysiology – Risk Factors and Potential Mechanisms.

Familial clusters of NMS along with an approximately 30-fold increase in recurrence compared with first-episode risk suggest a genetic predisposition to the disorder.^16,24,25^ Carriage of the A1 allele of the dopamine D2 receptor (*DRD2*) gene has been associated with low density of dopamine D2 receptors in the brain, mostly on the corpus striatum on the caudate region. Carriers of the A1 allele have a 10.5 times higher risk of developing NMS than noncarriers.^26^

The second hypothesis suggests that the primary defect in skeletal muscle arises from the direct toxic effects of pharmacological compounds on the musculoskeletal fibres. This theory is based on the clinical overlap between NMS and malignant hyperthermia, the efficacy of dantrolene, a hyantoin derivative, in treating NMS, and the known impact of antipsychotic drugs on calcium regulation in musculoskeletal fibers.^27^ Massive calcium influx into the musculoskeletal fibres leads to sustained contraction and rigidity. Supporting this, several in vitro studies have demonstrated that typical antipsychotics, such as chlorpromazine and fluphenazine, enhance calcium entry into the sarcoplasmic reticulum.^28^

### Clinical Presentation

NMS is characterized by a tetrad of classic clinical features including hyperthermia, extrapyramidal symptoms (the hallmark being rigidity), mental status changes, and autonomic instability with sympathetic overactivity (Figure 2).^3^ In the typical pattern of symptom evolution, mental status changes manifest first, followed by rigidity, then fever, and finally, autonomic dysfunction.^29^ Mental status changes classically take the form of delirium with fluctuating levels of consciousness, disorientation, and psychomotor agitation. However, patients can also present with catatonic signs that advance to severe encephalopathy characterized by stupor and coma.^30^ In addition to rigidity, other extrapyramidal features can include tremor (similar to Parkinson's disease), chorea, oculogyric crises (gaze may be fixated upwards), dystonia, opisthotonus, and trismus.^31^ Fever, although frequently high-grade and greater than 40 °C, may be a less consistent feature with second-generation antipsychotic agents.^32^ It is usually poorly responsive to conventional antipyretic drugs. Tachycardia, labile or high blood pressure, arrhythmias, and diaphoresis can all occur as part of the autonomic dysfunction.^31^

**Figure 2.**
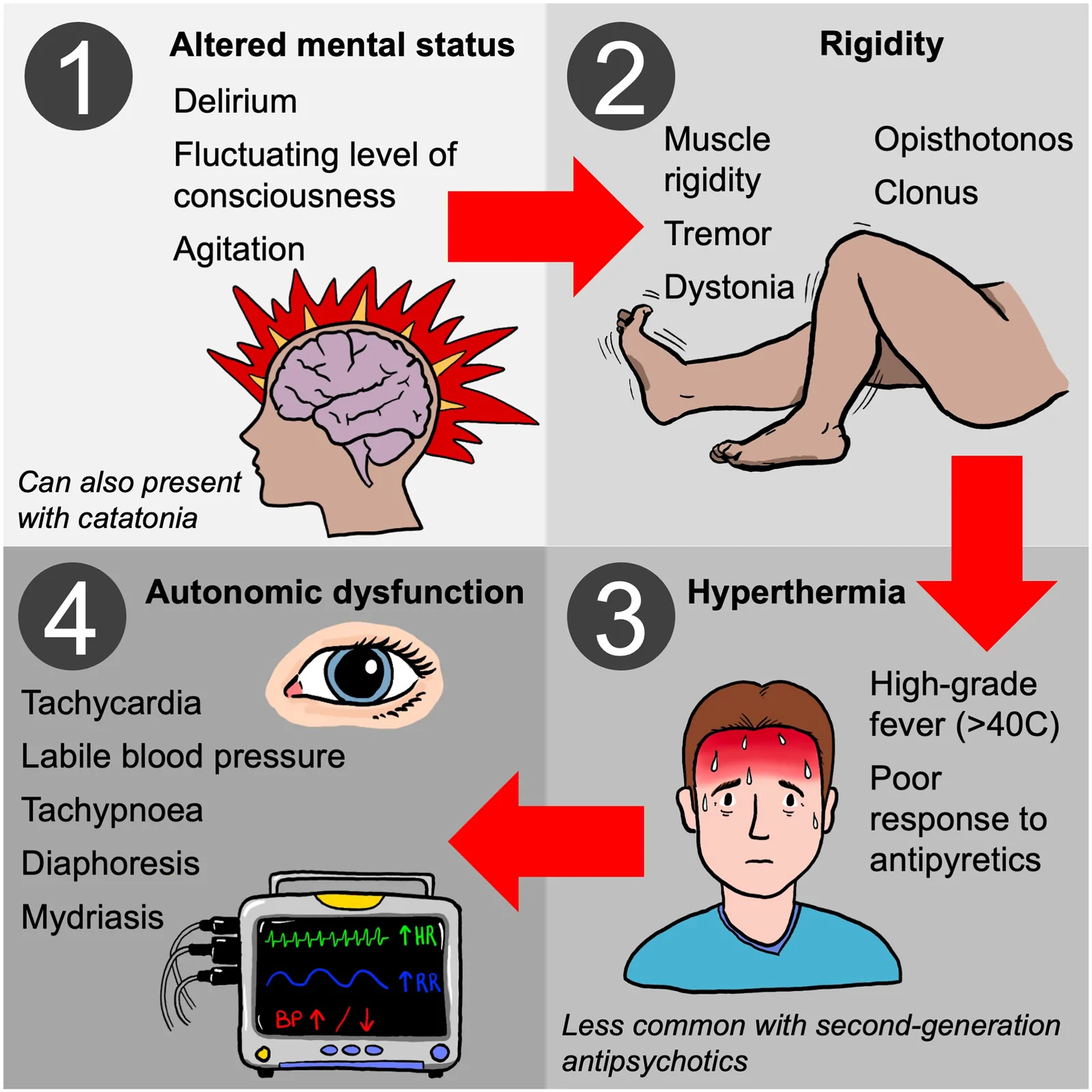
Clinical Presentation of Neuroleptic Malignant Syndrome.

In NMS, creatine kinase (CK) is usually greater than 1000 international units/L and the level typically correlates with severity and prognosis.^33^ Other laboratory abnormalities include a leucocytosis, electrolyte disarray (hypocalcemia, hypomagnesemia, hypo- and hypernatremia, hyperkalemia, and metabolic acidosis), acute kidney injury (AKI) from rhabdomyolysis, and low serum iron.

### Assessment and Diagnosis

NMS is diagnosed in the context of a typical clinical syndrome in the presence of an associated mediation. Although there is no gold-standard diagnostic test, the DSM-V diagnostic criteria can aid in making the diagnosis as outlined in Table 1. Neuroimaging and CSF analysis may be required to exclude alternative diagnoses such as structural brain disease or intracranial infection.^34^ MRI and CT are typically normal but occasionally generalised brain oedema or restricted diffusion in the cerebellum and basal ganglia have been reported.^35,36^ CSF protein is elevated in some cases.^24^ Electroencephalography (EEG) may be needed to rule out status epilepticus and only generalised slow wave activity is seen in NMS.

**Table 1.** Investigations and Diagnostic Criteria for Neuroleptic Malignant Syndrome.

Work-up	DSM-5 Diagnostic Criteria (2013)
CBC & differential Urea, creatinine & electrolytes Liver function tests Serial CPK Thyroid function tests Toxicology screen Blood cultures Urinalysis Brain imaging (CT or MRI) +/- lumbar puncture +/- EEG	1. Hyperthermia (oral temperature >38.0 °C on at least 2 occasions) 2. Rigidity 3. CPK >4-times the upper limit 4. Changes in mental status (delirium, altered consciousness) 5. Autonomic activation, including: tachycardia (>25% above baseline), diaphoresis, blood pressure elevation (systolic or diastolic ≥25% above baseline), or fluctuation (≥20mm Hg diastolic change or ≥25mm Hg systolic change), urinary incontinence, pallor, tachypnea (>50% above baseline)

### Treatment and Prognosis

The most critical step is to discontinue all antipsychotic medications. If feasible, other potentially contributing psychotropic drugs, such as lithium, anticholinergic therapies, and serotonergic agents, should also be stopped. It is important to be vigilant for sequelae including volume depletion, electrolyte disarray, AKI, sepsis, arrhythmias, cardiomyopathy, seizures, respiratory or liver failure, disseminated intravascular coagulation (DIC), and deep vein thrombosis (DVT).^37^

After discontinuation of antipsychotic or other precipitating agents, supportive care is then the mainstay of treatment of NMS, focused on managing the rigidity and fever and preventing the ensuing complications. Transfer to an intensive care unit (ICU) for monitoring and appropriate cardiorespiratory support is recommended. Volume depletion and hypotension should be with intravenous fluids (IVF). Antiarrhythmic agents, or temporary pacemakers may be required for arrhythmias.^38^ Hyperthermia can be managed with ice packs, cooling blankets, cooled IVF, and surface cooling devices. Antipyretics are generally ineffective. Aggressive fluid resuscitation and alkalization of urine with IV sodium bicarbonate may be needed to treat rhabdomyolysis and prevent AKI.^39^ Prophylactic intubation should be considered for patients experiencing excessive salivation, swallowing difficulties, coma, progressive respiratory failure, or severe rigidity. Use low-dose benzodiazepines to treat agitation and prescribe low-molecular weight heparin to prevent VTE.

Use of specific antidotes such as dantrolene or dopaminergic agents (bromocriptine or amantadine) in the management of NMS is controversial as the evidence of their benefit is conflicting and based only on case series.^40^ They are typically only employed in moderate or severe cases. One algorithm suggests starting with benzodiazepines (eg Diazepam 10-20 mg or lorazepam 1-2 mg every 4-6 h) and dantrolene (1-2.5 mg/kg q6-8hr hours IV with a maximal daily dose of 10 mg/kg/day), followed by the addition of bromocriptine (Starting dose 2.5 mg PO every 8 h; titrating up to 5-10 mg every 6-8 h). or amantadine (100 mg every 8 to 12 h; maximum dose 200 mg twice daily) if required (Table 2).^41,42^ Amantadine can be used as an alternative to bromocriptine as it has both dopaminergic and anticholinergic effects. Others advocate for first-line treatment with dopaminergic agents followed by the addition of dantrolene then if necessary in moderate to severe cases. Central alpha 2 agonists such as dexmedetomidine or clonidine may also be helpful adjuncts in managing agitation.

**Table 2.** Pharmacologic Management of Hyperthermic-Rigid Syndromes: Drug Characteristics and Clinical Considerations.

Drug	Mechanism of Action	Indications	Starting Dose	Dose Titration	Monitoring	Side Effects	Taper Required
Benzodiazepines	Enhances GABA-A receptor activity, promoting sedation and muscle relaxation	Agitation, sedation, seizures in NMS, serotonin syndrome, sympathomimetic toxidromes etc	Lorazepam 1–2 mg IV q4–6 h or Diazepam 5–10 mg IV	As needed for symptom control	Sedation level, respiratory rate, vital signs	Respiratory depression, sedation, confusion	Yes, if used long-term
Dantrolene	Inhibits calcium release from the sarcoplasmic reticulum in skeletal muscle	NMS, Malignant Hyperthermia, Baclofen withdrawal	NMS: 1–2.5 mg/kg IV q6–8h MH: 2.5 mg/kg IV initially, then 1–2.5 mg/kg IV every 5 min. Maintain with 1 mg/kg IV every 4–6 h until patient improving.	Max 10 mg/kg/day	Liver function, temperature, muscle rigidity	Hepatotoxicity, muscle weakness	Yes, over 10 days in some protocols
Bromocriptine	Dopamine agonist acting on D2 receptors to restore dopaminergic tone	NMS, Parkinsonism-Hyperpyrexia Syndrome	2.5 mg PO q8h	Up to 5–10 mg q6–8h	Mental status, blood pressure	Hypotension, nausea, hallucinations	Yes
Amantadine	NMDA receptor antagonist and weak dopamine agonist with anticholinergic effects	NMS, Malignant Catatonia (off-label)	100 mg PO q8–12h	Up to 200 mg BID	Mental status, renal function	Hallucinations, orthostatic hypotension	Yes
Dexmedetomidine	Alpha-2 adrenergic receptor agonist reducing sympathetic tone and agitation	Adjunct for sedation in NMS, serotonin syndrome, catatonia	0.2–1.5 mcg/kg/h IV infusion	Titrate to sedation goal	Heart rate, blood pressure	Bradycardia, hypotension	No
Clonidine	Alpha-2 adrenergic agonist reducing sympathetic outflow and BP	Adjunct in NMS, baclofen withdrawal	0.1 mg PO BID	Up to 0.3–0.6 mg/day	Blood pressure, heart rate	Dry mouth, hypotension, sedation	Yes
Cyproheptadine	Serotonin antagonist at 5-HT1A and 5-HT2A receptors	Serotonin Syndrome	12 mg PO loading, then 4–8 mg q6h	As needed for symptom control. Do not exceed 32 mg per 24 h.	Mental status, vital signs	Sedation, anticholinergic effects	No
Apomorphine	Dopamine agonist acting on D1 and D2 receptors	PHS when enteral route unavailable	2 mg SC q3h	The maximum recommended total daily dose of apomorphine is 20 mg, and no more than five doses per day should be administered.	Blood pressure, mental status	Nausea, hypotension	Yes
Rotigotine	Non-ergoline dopamine agonist delivered transdermally	PHS when enteral route unavailable	2 mg/24 h transdermal patch	Increase in 2 mg increments at weekly intervals based on response and tolerability. Max daily dose is 8 mg/24 h.	Skin reactions, mental status	Application site reactions, nausea, vomiting, orthostatic hypotension dizziness, hallucinations.	Yes
Baclofen	GABA-B receptor agonist causing muscle relaxation	Spasticity; withdrawal can cause rigidity syndromes	Starting dose if de novo: Oral baclofen 5 mg TDS Oral baclofen withdrawal: 5–10 mg orally TDS; For severe symptoms, start closer to 10 mg TDS Intrathecal baclofen withdrawal: Restart intrathecal infusion at the previous dose, if known; If pump malfunction prevents infusion: Temporary oral baclofen can be used: start at 10 mg TDS	Increase by 5 mg per dose every 3 days if needed Usual maintenance dose: 40–80 mg/day, divided TID	Muscle tone, mental status	Sedation, dizziness, weakness *Avoid if impaired kidney function as it can accumulate and cause profound sedation or coma*	Yes
Physostigmine	Reversible acetylcholinesterase inhibitor increasing acetylcholine levels	Central anticholinergic syndrome	0.5–2 mg IV slowly over 5 min	Repeat every 10–15 min if needed; some protocols allow up to 4 mg in 1 h	Continuous cardiac monitoring	Bradyarrhythmias, hypersalivation, bronchospasm, or seizures.	No

By inhibiting calcium release from the sarcoplasmic reticulum, dantrolene is a direct-acting muscle relaxant that reduces heat production and rigidity with effects reported within minutes of administration.^43,44^ It appears to shorten duration of illness with improvement in fever and rigidity trends noted over 1-2 days. There is a potential risk for liver toxicity and thus, dantrolene should generally be withheld if liver function tests are very abnormal. Some advocate for a subsequent slow taper of dantrolene or dopaminergic agents over 10 days to minimize risk of relapse; however, practices vary in this regard.^45^

In very severe cases requiring invasive mechanical ventilation, use of nondepolarizing neuromuscular blocking agents (eg, atracurium) may be considered to help achieve rapid control of rigidity and hyperthermia. Avoid antimuscarinic agents which may worsen hyperthermia.

The patient's underlying psychiatric condition must be assessed and managed during withdrawal of the antipsychotic therapy. Electroconvulsive therapy (ECT) may assist in regulating temperature, improving consciousness, and reducing diaphoresis in NMS, particularly in cases where the underlying psychiatric diagnosis is psychotic depression or catatonia, but it would be safer to use after the acute episode has resolved.^46^ A retrospective case series of 15 patients with refractory NMS who were treated with ECT over a 17-year period reported a remission rate of 73.3%.^47^ However, there is a risk of cardiac arrythmias and arrest after ECT.

Symptoms of NMS typically improve after discontinuing the antipsychotic, with most cases resolving within 1-2 weeks, though episodes triggered by long-acting depot injections may last up to a month. However, NMS can be life-threatening with mortality rates between 3% and 38% if not promptly recognized and treated.^48,49^ Complications such as severe rhabdomyolysis, AKI, and respiratory failure contribute to a poorer prognosis.^49^ Additionally, the underlying conditions for which neuroleptic medications were prescribed, particularly alcohol and drug addiction, can also influence the overall prognosis.

Clinicians should carefully review the patient's history for prior NMS episodes before prescribing new neuroleptic medications, as those patients are at higher risk of recurrence. Since NMS usually develops during dosage increases, both the clinician and the patient's family should remain vigilant until a stable dose is reached.

## Serotonin Syndrome

### Epidemiology

Serotonin syndrome is a life-threatening, drug-induced toxidrome triggered by serotonergic medications, resulting in excessive activation of central and peripheral serotonin postsynaptic receptors. This condition is characterized by a classic triad of mental status changes, autonomic hyperactivity, and neuromuscular abnormalities.^50^ It can occur in the context of therapeutic medication use, inadvertent drug interactions, intentional self-poisoning or in instances of kidney dysfunction causing accumulation of serotonergic medications.^51,52^

Serotonin syndrome can affect people of all ages and is increasingly reported more with growing use of serotonergic medications.^53^ Selective serotonin reuptake inhibitors (SSRIs) are frequently implicated, with tens of thousands of exposures reported annually in the United States.^54^ While SSRIs are less likely to cause severe serotonin syndrome compared to medications like monoamine oxidase inhibitors (MAOIs), serotonin-norepinephrine reuptake inhibitors (SNRIs) slightly increase the risk.^55^

### Pathophysiology

Serotonin, also known as 5-hydroxytryptamine (5-HT), serves as a mediator both in the peripheral and central nervous systems.^56^ In the periphery, it regulates various functions like vasoconstriction, uterine contraction, bronchoconstriction, gastrointestinal motility, and platelet aggregation. In the brain, serotonin modulates wakefulness, attention, mood, appetite, thermoregulation, and motor tone. Serotonin syndrome occurs when there's excessive stimulation of serotonin receptors due to elevated synaptic serotonin levels, typically triggered by certain medications. Although no single receptor is solely responsible, subtypes like 5-HT1A and 5-HT2A may play key roles.^52^ Most cases result from drug interactions rather than a single medication. Examples of agents that can precipitate serotonin syndrome are summarized in Table 3.

**Table 3.** Medications That can Precipitate Serotonin Syndrome and Their Mechanism of Action.

Mechanism of action	Medications
Increases serotonin formation	Tryptophan Oxitriptan
Increases release of serotonin	Amphetamines (including dextroamphetamine, methamphetamine) MDMA (Ecstasy) Amphetamine derivatives (eg, fenfluramine, dexfenfluramine, phentermine) Cocaine Mirtazepine
Inhibition of serotonin reuptake	Selective serotonin reuptake inhibitors (SSRIs) Serotonin-norepinephrine reuptake inhibitors (SNRIs) Tricyclic antidepressants (TCAs) Serotonin modulators (eg, trazodone, nefazodone) Serotonin reuptake enhancers (eg, dextromethorphan) Amphetamines Cocaine MDMA (Ecstasy)
Direct serotonin agonists	Buspirone Triptans (eg, sumatriptan) Ondansetron Mirtazapine Linezolid (antibiotic) Tryptophan
Inhibition of serotonin metabolism	Monoamine oxidase inhibitors (MAOIs) Serotonin precursors (eg, L-tryptophan) St. John's Wort
Increases sensitivity of postsynaptic serotonin receptor	Lithium

Serotonin syndrome can also be triggered by non-serotonergic agents which inhibit the metabolism of serotonergic drugs. Examples include the inhibition of CYP3A4 which normally metabolizes methadone, venlafaxine and oxycodone by drugs such as ciprofloxacin or ritonavir, or the inhibition of CYP2C19 which normally metabolises sertraline by fluconazole.

### Clinical Presentation

Serotonin syndrome encompasses a range of symptoms, with the intensity reflecting the degree of serotonergic activity.^57^ The typical triad of findings include mental status change, autonomic instability, and neuromuscular hyperactivity. Mental status changes may include anxiety, restlessness, agitated delirium, and even coma. Autonomic instability can present as tachycardia, hypertension, fever, flushing, diaphoresis, mydriasis, nausea, vomiting and diarrhoea. Manifestations of neuromuscular hyperactivity may include hyperreflexia, clonus, myoclonus, tremor, akathisia and later rigidity. Clonus, particularly bilateral ankle clonus, is a key indicator, but its absence doesn't rule out the syndrome as it may be difficult to appreciate in the presence of tremor.^58^ Hyperthermia, seizures, and high CK are adverse prognostic features.^59^

The syndrome typically develops promptly within 24 h of medication exposure or dose adjustment and will generally resolve within 24 h of discontinuing the causative drugs.^60^ The rapid onset and offset can be helpful to differentiate serotonin syndrome from various other similar disorders including NMS and thyrotoxicosis.

Laboratory tests may show nonspecific findings like leucocytosis and elevated CPK levels, but there's no specific diagnostic test for serotonin syndrome. In severe cases, there is a risk of systemic complications including DIC, rhabdomyolysis, metabolic acidosis, AKI, and acute respiratory distress syndrome (ARDS).^60^

### Assessment and Diagnosis

Serotonin syndrome is diagnosed clinically, typically using the Hunter Toxicity Criteria Decision Rules which seem to be the most accurate of the available diagnostic criteria with a sensitivity and specificity of 84% and 97%, respectively (Figure 3).^61^ Serotonin syndrome is uncommon in patients on stable doses of serotonergic drugs and is typically precipitated by overdose, drug interactions, recent initiation or dose increase of such medications.

**Figure 3.**
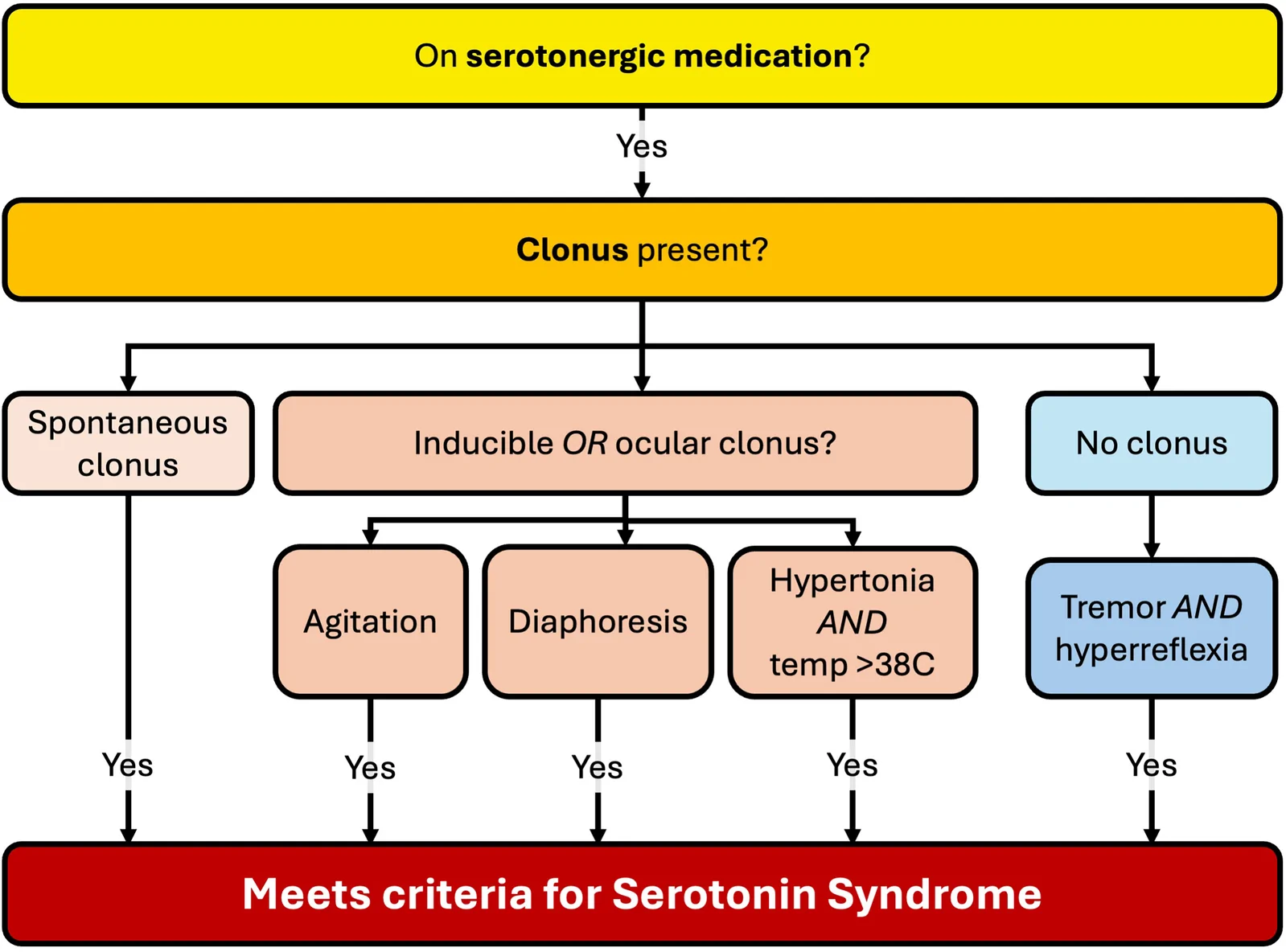
The Hunter Diagnostic Criteria for Serotonin Syndrome.

### Treatment

Management of serotonin syndrome varies by severity of case but typically involves discontinuation of serotonergic agents, supportive care, sedation with benzodiazepines, consideration of the need for serotonin antagonists such as cyproheptadine, and evaluation of the need to restart serotonergic drugs after symptom resolution.

Supportive care is the mainstay of treatment and involves supplemental oxygen, continuous cardiac monitoring and administering IV fluids. Benzodiazepines (eg IV lorazepam 2-4 mg or diazepam 5-10 mg) are used for sedation in agitated patients. Physical restraint should be avoided where possible as it may result in isometric muscle contractions leading to severe lactic acidosis and worsening fever.^53^ Autonomic instability resulting in hypertension may require treatment with short-acting parenteral agents (eg esmolol or nitroglycerine infusion) to control blood pressure. In patients with hypotension from MAOIs, consider low doses of direct-acting sympathomimetic agents such as phenylephrine, epinephrine, or norepinephrine. Avoid opioids (including fentanyl and oxycodone) and hydralazine which may increase serotonin levels.

Hyperthermia control is crucial as this may precipitate rhabdomyolysis, seizures, metabolic acidosis, and DIC.^62^ Patients whose temperature is > 41.1 °C require immediate sedation, neuromuscular paralysis with non-depolarizing agents, and intubation along with external cooling measures (eg cooling blanket, Arctic Sun). Antipyretics such as paracetamol are not effective as the mechanism of hyperthermia relates to excessive muscular activity as opposed to a change in the hypothalamic temperature set point.

Cyproheptadine may be used as an antidote if benzodiazepines and supportive care fail (Table 2).^63^ It is a first-generation, sedating histamine-1 receptor antagonist with nonspecific 5-HT1A and 5-HT2A antagonistic activity. The loading dose is 12 mg, followed by a maintenance dose of 4–8 mg every 6 h. It can be administered orally or via nasogastric tube. Potential side effects include hypotension and anticholinergic symptoms (eg tachycardia, urinary retention, exacerbation of hyperthermia). Its gradual onset of action limits its efficacy and often it is not required because serotonin syndrome may resolve spontaneously within 24 h. Other antidotes like antipsychotic agents and bromocriptine are not recommended due to limited efficacy or potential exacerbation of symptoms.

Dexmedetomidine is a newer sedative that may offer specific, mechanistic benefits in serotonin syndrome due to its stimulation of alpha-2C receptors in the striatum which appears to inhibit serotonin release (Table 2).^64^ Furthermore, it stimulates alpha-2A receptors in the prefrontal cortex and locus coeruleus, decreasing agitation and sympathetic tone. Case reports suggest that it may be particularly useful for patients who are refractory to other sedatives or where you wish to avoid intubation.^65^ However, benzodiazepines are still preferred in cases where there have been seizures or where additional anti-epileptic activity is required.

### Prognosis

Serotonin syndrome typically resolves rapidly once causative medications are discontinued, often within a day.^53^ Patients primarily require supportive care, and recovery is expected with cessation of offending drugs. Severe cases necessitate intensive care, while moderate cases warrant hospitalization for observation. Symptoms usually resolve within 24 h, but medications with long durations of action may prolong recovery.

## Malignant Hyperthermia

### Epidemiology

Malignant hyperthermia (MH) is a rare hypermetabolic crisis caused by any inhalational anaesthetic agent (eg sevoflurane, isoflurane) or depolarizing muscle relaxant (specifically succinylcholine) in a genetically susceptible individual.^66^ The prevalence of MH is estimated to be 1 in 100 000 administered anaesthetics.^67^ However, this may be an underestimate due to the variable penetrance of the underlying genetic mutations. MH events typically occur during the operation or within the first hour after anaesthesia ends.^68^ Patients with central core myopathy are also vulnerable to developing these episodes. MH events are more common in males than females (2:1) and approximately half of all reported events occur in children.^69^

### Pathophysiology

MH-susceptible individuals harbour genetic skeletal muscle receptor anomalies that permit excessive myoplasmic calcium accumulation upon exposure to certain anaesthetic triggering agents.^70^ The precise mechanism of anaesthetic interaction with these aberrant receptors, precipitating an MH crisis, remains undefined but is likely linked to compromised magnesium inhibition of sarcoplasmic reticulum calcium release and extracellular calcium influx.

During an MH episode, excessive calcium accumulation in skeletal muscle cells leads to prolonged muscle contractions, rhabdomyolysis, cellular hypermetabolism, and metabolic acidosis.^71^ In normal muscle physiology, depolarization spreads via the transverse tubule system, activating dihydropyridine receptors coupled to ryanodine receptors, facilitating calcium passage into the intracellular space, leading to muscle contraction. Conversely, in MH physiology, mutations in RYR1 or DHP receptors result in uncontrolled calcium release from the sarcoplasmic reticulum and transsarcolemmal calcium influx via TRPC channels, precipitating an acute MH crisis.^72^ The uncontrolled influx of myoplasmic calcium prompts sustained muscle contraction, hypermetabolism, and acidosis, culminating in rhabdomyolysis and hyperkalemia.^73^ Dantrolene, the sole therapy for MH, inhibits calcium release from the sarcoplasmic reticulum via RYR1 receptor binding, necessitating calmodulin presence and sufficient magnesium levels.^74^

MH can also manifest post-cardiopulmonary bypass or spontaneously in MHS individuals following heat exposure or exertion.^75,76^

### Clinical Presentation

Manifestations of MH vary among patients, and not all features may develop. It can appear shortly after anaesthesia induction or anytime during maintenance, even post-anesthesia. Initial signs include unexplained end-tidal CO2 increase, tachypnea, sinus tachycardia, and muscle rigidity.^69^ Hyperthermia may occur after hypercarbia and tachycardia, with accurate core temperature monitoring vital for diagnosis. The temperature can rise rapidly by 1-2 °C every five minutes. Generalized muscle rigidity, often despite neuromuscular blockade, and masseter muscle rigidity may occur. ECG abnormalities can occur due to hyperkalemia. Myoglobinuria, indicated by dark urine, and other lab findings including mixed metabolic and respiratory acidosis, hyperkalemia, elevated CK and DIC may accompany MH episodes.^69^

Postoperatively, some patients may experience isolated rhabdomyolysis without classic MH signs.^77^ Recrudescence, seen in about 20%, presents with elevated temperature and organ failure risks.^78^

### Assessment and Diagnosis

MH is suspected when end-tidal CO_2_ rises despite increased ventilation, supported by muscle rigidity or metabolic acidosis. Diagnosis during an acute episode is presumptive based on typical clinical signs; family history or prior anaesthetics do not always correlate. Treatment should commence urgently upon suspicion. Laboratory tests, while supportive, are not mandatory for diagnosis. Post-event, MH likelihood can be gauged using the MH clinical grading scale, but definitive diagnosis requires susceptibility testing.

### Treatment

The initial emergency response should involve activation of a dedicated MH cart if available or else contact pharmacy for required medications and equipment (ie, dantrolene, IV calcium, IV insulin, D50W, isotonic bicarbonate, cooled crystalloids). There may be a national MH hotline available for additional guidance. Immediate actions should include cessation of the surgical procedure and elimination of any residual volatile anaesthetic by hyperventilation with 100% oxygen at 2-3 times the normal respiratory rate or insertion of activated charcoal filters into the inspiratory and expiratory limbs of the anaesthesia circuit. Adjust the minute ventilation parameters in order to achieve an end-tidal pCO2 of approximately 25 mm Hg (3.3 kPa), if feasible.

Administer dantrolene 2.5 mg/kg IV initially, then 1-2.5 mg/kg IV every 5 min (Table 2). Maintain with 1 mg/kg IV every 4-6 h until the patient has been stable and improving for 24 h. Continuously monitor core temperature and initiate cooling if >39 °C. Utilize various cooling techniques such as ice packs, evaporative cooling, cooling blankets, cooled fluids, cold lavage of the surgical field, surface cooling devices (eg Arctic Sun) as needed. Cease cooling once temperature falls below <38 °C.

Treat hyperkalemia using IV calcium, insulin, D50W, and isotonic bicarbonate fluid resuscitation. Consider beta-2 agonists for refractory cases. For rhabdomyolysis, give fluid resuscitation with crytalloids or isotonic bicarbonate and aim for urine output >1 ml/kg/hour. Monitor for any symptoms/signs of compartment syndrome.

### Prognosis

Based on data from the National Inpatient Sample and the North American Malignant Hyperthermia Registry, mortality from MH has significantly reduced due to ETCO_2_ monitoring and dantrolene availability, now estimated 6–10%.^79,80^

Core temperature monitoring is crucial in reducing MH mortality as skin temperature doesn't reliably reflect core temperature.^69^ Continuous core temperature monitoring aids in quicker diagnosis and treatment, reducing peak temperature and duration of hyperthermia. Analysis from Malignant Hyperthermia Association of the United States (MHAUS) shows patients without temperature monitoring were twice as likely to die, and those with skin temperature monitoring were 1.5 times as likely, compared to core temperature monitoring.^79^ Deaths mostly occurred with peak temperatures ≥38.9 °C. Other risk factors for cardiac arrest include older age, multimorbidity, high muscle mass and the development of DIC.^80^

## Parkinsonism-Hyperpyrexia Syndrome

### Epidemiology

Parkinsonism-hyperpyrexia syndrome (PHS) is considered to be a form of NMS that occurs upon rapid discontinuation of treatment for Parkinson's disease (PD).^51^ The first documented case dates back to 1981, involving a 63-year-old woman who exhibited altered consciousness, diaphoresis, fever, and elevated serum CK after stopping levodopa, amantadine, and biperiden.^81^ Similar clinical presentations have been reported under various names like “lethal hyperthermia” and “dopaminergic malignant syndrome,” but the consensus term is now PHS. The rarity of PHS is attributed to the specific circumstances leading to its occurrence, primarily the sudden withdrawal of antiparkinsonian drugs in patients with PD. Literature on PHS mainly consists of case reports,^82^ revealing a higher incidence among males than females. The duration of parkinsonism varies from 2 to 16 years, with baseline levodopa dosages ranging from 200 to 2100 mg per day.

### Pathophysiology

Studies suggest that reduced levels of dopamine metabolites and increased levels of noradrenaline metabolites in the CSF of PD patients who have experienced NMS-like episodes indicate a role of central dopaminergic hypoactivity and noradrenergic hyperactivity in the development of NMS, particularly in elderly PD patients.^83^

Historically, the sudden reduction or cessation of dopaminergic medications, particularly during outdated practices like “drug holidays” for PD patients with psychosis, may have triggered many previously documented cases of PHS.^81^ Discontinuation, changing, or adjusting doses of any antiparkinsonian drugs have been linked to PHS occurrences. These scenarios may occur in the context of swallowing difficulties, fasting, or inappropriately holding medications prior to a procedure/surgery. Switching from immediate-release to extended release formulations, infection, volume depletion, or dysfunction of a deep brain stimulator are also other potential triggers.^84^ Additionally, medications such as tolcapone, which is a catechol-O-methyltransferase inhibitor used to prolong levodopa's effects by reducing its peripheral breakdown, have also been associated with PHS despite lacking direct dopaminergic action.^85^ Factors such as longer duration of PD, motor fluctuations (which often accompany advanced PD stages), psychiatric comorbidities, and older age are correlated with an increased risk of developing PHS.

### Clinical Presentation

The clinical presentation of PHS resembles that of NMS but extrapyramidal signs (eg, tremor, rigidity). tend to predominate^51^ Symptoms typically emerge within 18 h to 7 days following a change in dopaminergic medication which represents a longer latent interval than that compared to NMS.^86^ Rigidity and tremor are often the first symptoms followed by akinesia, hyperpyrexia, agitation, delirium, stupor, and coma. Dysautonomic features may also be present including tachycardia, labile BP and diaphoresis. Myoclonus and seizures can also occur. Laboratory abnormalities include an elevated CK and leucocytosis, although these tend to less prominent compared to NMS. Elevated liver transaminases and metabolic acidosis may also be observed.

### Assessment and Diagnosis

The diagnosis of PHS is often one of exclusion and the clinical diagnostic criteria used for NMS may be helpful. Look for suspicious contexts such as missed doses, fever of unknown origin, inadvertent discontinuation or delayed antiparkinsonian treatment when PD patients are hospitalized for other reasons. The most consistent features of PHS include rigidity, hyperpyrexia, and altered consciousness.^83^ Evaluation for other toxic-metabolic encephalopathies and hyperpyrexia syndromes is necessary to rule out alternative diagnoses.

Dyskinesia-hyperpyrexia syndrome is an important, albeit rare differential diagnosis to consider in these cases.^82^ This syndrome can result following an overdose of dopaminergic medication or occasionally due to a physiological stressor (eg, infection, volume depletion, heat stroke). These patients present with dyskinesia, rhabdomyolysis, hyperthermia, and altered mental status. Treatment is usually supportive along with careful down-titration of PD therapies.^87^

### Treatment and Prognosis

PHS is a neurological emergency necessitating intensive care management. Treatment involves similar supportive measures as for NMS, including cooling, IV fluids, and antihypertensives. Reinstitution of dopaminergic therapies such as levodopa and bromocriptine are essential.^83^ Nasogastric tube insertion may be necessary to administer these therapies. In patients in whom an enteral route is contraindicated, a transdermal or parenteral dopamine agonist may be required (eg, apomorphine 2 mg SC q3hr, or rotigotine) (Table 2). If a deep brain stimulator is present, it should be checked to ensure that it is functioning correctly.

Parkinsonian patients often have abnormal gastrointestinal absorption, especially in an intensive care setting, due to altered intestinal motility. Therefore, careful consideration is needed when administering levodopa. It is recommended to start bromocriptine at a dose of 5 to 10 mg per day, divided into three doses, and adjust the dose as needed.^88^ Levodopa should be administered at the patient's previous dose and modified as necessary. In general, PHS tends to resolve more quickly and has a better prognosis than NMS.^86^ However, PD patients who experience NMS-like episodes may have worse disability outcomes than those who do not.^89^

### Malignant Catatonia

Catatonia is a motor dysregulation syndrome due to dysfunction of the basal ganglia involving difficulty initiating or terminating actions.^90^ While catatonia has traditionally been linked with schizophrenia, it is frequently precipitated by acute medical conditions or mood disorders such as bipolar disorder. Potential aetiologies include drugs/toxins (eg, antipsychotics, lithium toxicity, steroids, cocaine withdrawal), metabolic conditions (eg, DKA, adrenal insufficiency, uraemia), structural brain diseases (eg, tumour, ICH, stroke), CNS infection/inflammation (eg, meningitis, encephalitis, autoimmune encephalitis), ictal/postictal states and psychiatric disorders (eg, affective disorders, psychotic disorders, autism spectrum disorder). Identifying catatonia in psychiatric settings poses challenges due to the potential overlap in symptoms between catatonia and other psychiatric conditions. It can be diagnosed in roughly 10% of acutely hospitalized psychiatric or medical patients, with greater prevalence among those being evaluated for delirium.^91^

Emerging evidence suggests a multifactorial etiology involving dysregulation of neurotransmitter systems, with inadequate dopamine activity at D2-receptors, inadequate gamma-aminobutyric acid (GABA) signalling and excessive signalling via glutaminergic NMDA receptors, along with immune-mediated mechanisms.^92,93^

There are two main types of catatonia - hypokinetic (causing stupor) or hyperkinetic (causing agitation), with the former being more common though patients can cycle between the two extremes. Malignant catatonia is a severe form that can complicate either hypokinetic or hyperkinetic catatonia. It is characterised by catatonia with autonomic instability (including hyperthermia which is a prerequisite for the diagnosis). Other autonomic features include diaphoresis, tachycardia, and labile BP. Rigidity is found in 80% of patients along with altered mental status.^93^ This condition poses a significant clinical challenge due to its potential to rapidly escalate to life-threatening complications, including catatonic stupor.

Management typically involves a combination of pharmacotherapy, including benzodiazepines (eg, lorazepam 2 mg IV q8hr) and ECT, alongside supportive care. Treat any underlying aetiologies and avoid anti-dopaminergic agents, especially antipsychotics in those who are treatment naïve.^94^ Dexmedetomidine and valproate may be useful in agitation.^95,96^ Daily ECT for 2-5 days may be needed in malignant catatonia, particularly if there has been an inadequate response to medical therapy or in those with underlying affective disorders.^90^ There is some evidence from case series to support the use of NMDA receptor antagonists such as amantadine or memantine.^97^ Recovery occurs over 4-10 days, during which time the benzodiazepines can be tapered and stopped.

### Other Drug-Related Syndromes

Other drug-related syndromes and toxidromes, such as baclofen withdrawal, central anticholinergic syndrome, and amphetamine toxicity, can present with clinical features that mimic NMS and other febrile encephalopathies with rigidity. Table 4 summarises the clinical features that can be used to help distinguish between these various syndromes and toxidromes. Baclofen withdrawal, characterized by abrupt cessation or rapid reduction of baclofen therapy, can lead to fever, rigidity, altered mental status, and autonomic dysfunction, mirroring NMS symptoms.^98^ There may be recrudescence of any underlying spasticity (rebound spasticity). Intrathecal baclofen withdrawal tends to be more severe than oral. Plain radiographs may be used to identify catheter kinking, migration or disconnection. Prompt resumption of oral or intrathecal baclofen is the mainstay of management. In the case of the latter, pump revision in the operating room may be required or else temporary measures such as infusion into a lumbar drain or a single bolus via lumbar puncture may be considered as bridging strategies.^99^ Multimodal therapy, utilizing agents like oral baclofen, benzodiazepines, central alpha-2 agonists (such as dexmedetomidine and tizanidine), cyproheptadine, and dantrolene, may be necessary to provide symptomatic relief while avoiding respiratory depression (Table 2).

**Table 4.** Distinguishing the various Aetiologies, Syndromes, and Toxidromes That can Cause Acute Febrile Encephalopathy with Rigidity.

Syndrome	Onset	Typical Clinical Findings	Laboratory Abnormalities	Causative Agents	Treatment Agents	Resolution
Neuroleptic Malignant Syndrome (NMS)	Within days to weeks after the initiation or dose escalation of neuroleptic medications	Lead-pipe rigidity, hyperthermia, altered mental status, autonomic dysfunction	Elevated CK levels +/- leucocytosis, electrolyte disarray, AKI	Antipsychotic medications	Discontinuation of offending agent, supportive care, benzodiazepines, dantrolene, bromocriptine, or amantadine	Improvement within days to weeks.
Serotonin Syndrome	Rapid, often within hours	Hyperreflexia, clonus, diaphoresis, myoclonus, hyperthermia, agitation, altered mental status	Elevated CK levels +/- leucocytosis, metabolic acidosis, AKI, DIC	Serotonergic medications or drug interactions	Discontinuation of offending agent, supportive care, benzodiazepines, cyproheptadine	Typically resolves rapidly once causative medications are discontinued, often within a day
Malignant Hyperthermia	Typically occurs during or after general anesthesia or administration of triggering agents, although rare spontaneous cases have been reported. Onset can be rapid, within minutes of exposure to triggering agents.	Rapid onset of hyperthermia, often exceeding 40 °C, along with muscle rigidity, tachycardia, tachypnea, cyanosis, and metabolic acidosis. Patients may exhibit signs of increased muscle metabolism such as muscle rigidity, masseter spasm, and generalized muscle contractions.	Elevated CKD, mixed acidosis, hyperkalemia, myoglobinuria.	Triggered by exposure to volatile anesthetic gases (eg, halothane, isoflurane, desflurane) or depolarizing muscle relaxants (eg, succinylcholine). Rarely, it can be triggered by stressors such as exercise, heat, or emotional trauma.	Immediate discontinuation of triggering agents. Administration of dantrolene, which inhibits calcium release from the sarcoplasmic reticulum, thereby reducing muscle contractility and metabolic activity. Supportive measures include cooling measures, intravenous fluids, and correction of electrolyte imbalances.	Prompt recognition and treatment can lead to rapid resolution of symptoms. With appropriate management, patients typically recover within hours to days.
Parkinsonism-Hyperpyrexia Syndrome	Abrupt, triggered by medication withdrawal	Rigidity, hyperthermia, altered mental status	Elevated CK, leucocytosis, (less prominent compared to NMS)	Dopaminergic medications	Reintroduction of dopaminergic therapy, supportive care	Gradual resolution of symptoms over days to weeks
Malignant Catatonia	Acute, rapid onset	Severe rigidity, hyperthermia, altered consciousness, autonomic instability	May show nonspecific abnormalities such as leukocytosis or elevated inflammatory markers	Various; may be associated with psychiatric disorders or acute stressors (eg drugs, infections, metabolic conditions)	Benzodiazepines, ECT, supportive care	Clinical improvement often seen within days to weeks with prompt treatment, though resolution may vary widely.
Baclofen Withdrawal	Develops within 1-4 days of stopping oral baclofen (or <48 h after stopping intrathecal baclofen)	Fever, rigidity, altered mental status, autonomic dysfunction	None specific	Baclofen therapy withdrawal (oral or intrathecal)	Reintroduction of baclofen, benzodiazepines, supportive care	Gradual resolution after re-instatement of the therapy.
Central Anticholinergic Syndrome	Rapid	Delirium, agitation, mydriasis, hallucinations, tachycardia, dry mouth, urinary retention, dry skin.	None specific	Anticholinergic medications (eg, scopolamine, atropine), certain antidepressants (eg, tricyclic antidepressants)	Discontinuation of offending agent, supportive care, physostigmine (in severe cases)	Gradual, with cessation of offending agent and supportive care
Sympathomimetic toxidrome	Rapid	Hypertension, tachycardia, dilated pupils, diaphoresis, agitation	Possible hyperglycemia, metabolic acidosis, leukocytosis	Stimulant drugs (eg, amphetamines, cocaine), certain antidepressants (eg, SNRIs)	Supportive care, benzodiazepines (for agitation and hypertension), cooling measures	Gradual with cessation of drug use and supportive care
Seizures	Rapid	Aura, altered consciousness, muscle contractions or rigidity, autonomic dysfunction, incontinence	EEG abnormalities, metabolic abnormalities - abnormal electrolytes, blood glucose, or toxicology screen may reveal aetiology of seizure where provoked.	Epilepsy, brain injury, drug intoxication, fever, infection	Antiepileptic drugs, benzodiazepines, supportive care.	Variable, depends on underlying cause and treatment efficacy.
Autoimmune/Paraneoplastic Encephalitis	Variable, often acute onset	Rapid cognitive decline, seizures, psychiatric symptoms	CSF pleocytosis, anti-neuronal antibodies, EEG abnormalities	Autoimmune disease or paraneoplastic syndrome, malignancy	Immunotherapy (eg, corticosteroids, IV immunoglobulins), tumor treatment	Variable, depends on underlying cause and treatment response.
CNS Infections	Variable, often acute onset	Headache, fever, altered mental status, focal neurological deficits	Elevated white blood cell count in CSF, positive CSF cultures (bacteria, viruses, fungi), imaging abnormalities (eg, abscess, ventriculitis)	Bacteria (eg, Streptococcus pneumoniae, Neisseria meningitidis), viruses (eg, herpes simplex virus, varicella-zoster virus), fungi (eg, Cryptococcus neoformans)	Antibiotics, antivirals, antifungals, supportive care	Variable, depends on the type and severity of infection, as well as the timeliness and efficacy of treatment

Central anticholinergic syndrome is a rare but potentially serious condition caused by the blockade of central muscarinic receptors in the brain due to the ingestion or exposure to anticholinergic medications or substances with anticholinergic activity (eg, scopolamine, doxylamine, tricyclic antidepressants, antipsychotics). 15–20% of admissions for acute poisoning may be caused by anticholinergic delirium.^100^ The syndrome typically presents with a wide range of symptoms including altered mental status, agitation, hallucinations, delirium, and autonomic instability such as hyperthermia, tachycardia, and urinary retention. Dry skin is an essential feature, which helps separate anticholinergic from sympathomimetic toxidromes. Diagnosis is primarily clinical and based on the patient's history of anticholinergic exposure along with characteristic signs and symptoms. Management involves discontinuation of the offending agent, supportive care to stabilize vital signs and hydration status, and in severe cases, administration of physostigmine, a cholinesterase inhibitor that reverses central anticholinergic effects.

Sympathomimetic toxidromes encompass a spectrum of signs and symptoms resulting from excessive stimulation of the sympathetic nervous system, commonly seen with the use of substances such as amphetamines, cocaine, or synthetic cathinones (“bath salts”). Epidemiologically, it affects a wide range of age groups, predominantly involving young adults. Pathogenesis involves the activation of adrenergic receptors, leading to increased catecholamine release and subsequent sympathetic activation. Clinically, patients typically present with agitation, tachycardia, hypertension, diaphoresis, mydriasis, hyperthermia, and sometimes seizures or hallucinations. Diagnosis is primarily clinical, based on the characteristic constellation of symptoms and history of substance use. Management involves supportive care to stabilize vital signs and prevent complications such as arrhythmias or hyperthermia.^101^ Benzodiazepines are often used to control agitation and seizures, while cooling measures may be necessary for hyperthermia. Additionally, titratable anti-antihypertensive infusions such as nitroglycerin or nicardipine may be required to control hypertension.

## Conclusions

In conclusion, this review highlights the complexity inherent in diagnosing and managing acute febrile encephalopathy with rigidity, encompassing conditions such as neuroleptic malignant syndrome, serotonin syndrome, and malignant hyperthermia. Careful risk stratification and psychotropic drug stewardship may help prevent or lead to earlier diagnosis of such cases. Further research is needed to unravel the underlying biological mechanisms and to identify the most effective treatment algorithms for managing many of these conditions.
